# Structured illumination for surface-resolved grazing-incidence X-ray scattering

**DOI:** 10.1107/S1600577526005977

**Published:** 2026-07-15

**Authors:** Doğa Gürsoy, Xiaogang Yang, Dina Sheyfer, Michael Wojcik, Ruipeng Li, Esther H. R. Tsai

**Affiliations:** a Advanced Photon Source Argonne National Laboratory USA; b National Synchrotron Light Source II Brookhaven National Laboratory USA; c Center for Functional Nanomaterials Brookhaven National Laboratory USA; Paul Scherrer Institute, Switzerland; EPFL, Switzerland

**Keywords:** structured illumination, coded aperture, synchrotron imaging, grazing angle, X-ray scattering

## Abstract

Grazing-incidence (GI) scattering techniques are widely used to characterize thin films, offering high surface sensitivity and insight into morphology and structure. However, these approaches typically provide statistical averaged information due to elongated footprint or limited spatial resolution due to beam size. Here we introduce a method that combines structured illumination with GI X-ray scattering and leverages our computational imaging approach to resolve local structural details. We demonstrate that our method captures local features of an organic semiconductor thin film without the need for sample rotation as in tomography. The method expands GI techniques from statistical averaging to high-resolution imaging, thereby providing the capability for detailed analysis of local material properties, such as domain shape, orientation and polymorphism, which are critical for advancing material design towards more efficient and tailored materials.

## Abstract 2

We introduce a method combining structured illumination with grazing-incidence X-ray scattering and computational imaging to resolve local structural details. Demonstrated on an organic semiconductor thin film, this method captures local features without sample rotation, enabling detailed analysis of material properties critical for advanced material design.

## 1. Introduction

X-ray scattering methods are widely employed for material characterization in a variety of fields, including chemistry, physics, materials science, and nanotechnology. These techniques are instrumental in probing the structural properties of different materials, providing critical insights into their morphology, phase, and crystalline properties. Among these, grazing-incidence (GI) X-ray scattering techniques, such as grazing-incidence wide-angle X-ray scattering (GIWAXS) and grazing-incidence small-angle X-ray scattering (GISAXS), are particularly effective for characterizing thin films, as they probe surface and near-surface regions without substrate limitations. These methods offer statistical information on crystalline domains and morphology, making them highly effective for investigating complex materials, including polymer blends, organic semiconductors, and self-assembled nanostructures. In particular, GIWAXS is used as a non-destructive tool for analyzing both in-plane and out-of-plane features, and plays a key role in monitoring real-time thin-film growth processes, offering crucial insights for optimizing materials in applications such as solar cells, transistors, and coatings (Lim *et al.*, 1987 ▸; Pietsch *et al.*, 2004 ▸; Stribeck, 2007 ▸; Zhang *et al.*, 2017 ▸; Müller-Buschbaum, 2014 ▸; Benvenuti *et al.*, 2023 ▸; Richter *et al.*, 2017 ▸; Sidhik *et al.*, 2024 ▸).

Despite its versatility, conventional GI X-ray scattering methods often face challenges in resolving individual domains, *e.g.* variation in orientation or crystallinity within or across domains, due to the extended X-ray footprint in grazing incidence geometry. To overcome these limitations, GI with micro-beam (Li *et al.*, 2012 ▸; Roth *et al.*, 2003 ▸) provides spatially resolved structural information, but its resolution is constrained by the focusing optics and the attainable beam size. Computed tomography (CT) with GISAXS or GIWAXS can be combined to reconstruct local variations in material properties (Kuhlmann *et al.*, 2009 ▸; Innis-Samson *et al.*, 2011 ▸; Ogawa *et al.*, 2015 ▸; Ogawa *et al.*, 2017 ▸; Ogawa *et al.*, 2020 ▸; Smilgies *et al.*, 2022 ▸) and domain orientations (Tsai *et al.*, 2021 ▸). However, CT-based approaches present practical challenges, particularly the requirement for sample rotation, which is susceptible to several issues, including runout errors, stage drift, and alignment difficulties, especially at tilted geometries. For *in situ* experiments, it is ideal and at times necessary to minimize sample movement and rotation because any small motions can cause complications with cabling. In tomography, long scanning times restrict the types of *in situ* studies that can be done and limit real-time visualization, while incomplete angular data, often due to beamline interruptions, can also introduce artifacts that degrade resolution.

**Figure 1:**
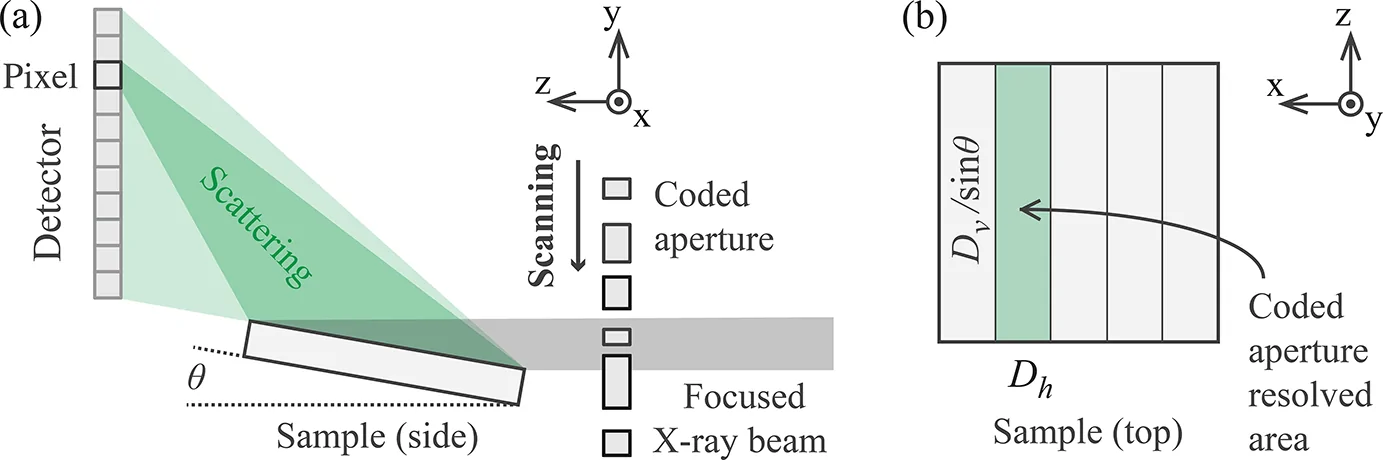
(*a*) Schematic of the SI-GID setup. A focused X-ray beam illuminates a sample at a grazing-incidence angle, with scattered signals recorded by a 2D pixel detector (*x*-*y* plane). A coded aperture structures the beam along the *y*-axis, and scanning in this direction encodes scattering signals for local sample variation along the *z*-axis. (*b*) Top view of the sample and the X-ray footprint (green), to be resolved in the *z*-axis via SI-GID.

To overcome these challenges, we propose a structured illumination technique (Forbes *et al.*, 2021 ▸) for grazing-incidence diffraction (SI-GID), which resolves local structural variations without requiring sample rotation. This method is compatible with existing sample environments and *in situ* experiments, providing a robust alternative to conventional approaches. As illustrated in Fig. 1 ▸, our SI-GID approach utilizes absorbing micro-coded apertures to structure the X-ray beam in this low-coherence setup, with the aperture scanned vertically along the *y*-axis to introduce measurement diversity.

For a beam with vertical and horizontal dimensions *D*_v_ and *D*_h_, respectively, the beam footprint extends along the *z*-axis as 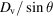 due to the small incidence angle. Meanwhile, the resolution along the translational *x*-axis is determined by the horizontal beam width *D*_h_. At each horizontal *x* position, sample local information can be extracted without sample rotation via computational reconstruction by scanning the 1D coded aperture. The method enables reconstruction of the scattering pattern for each sample pixel along the X-ray footprint (*z*-axis), as illustrated in Fig. 1 ▸(*b*). Unlike tomography, that necessitates complete tomographic data collection at all translational and rotational positions to achieve any spatial resolving power, our method enables imaging of specific regions of interest, offering flexibility to choose one or multiple translation positions. This approach avoids the compounding of multiple stage movement errors seen in tomography, where errors accumulate in the reconstruction.

## 2. Methods

### 2.1. Working principle

In the grazing-incidence measurement geometry, the X-ray beam probed an elongated region of the sample. Our approach employs a coded aperture to generate structured illumination, enabling selective probing of different sample regions. As the aperture is scanned vertically, distinct illumination patterns are produced, allowing different sets of sample regions to be probed.

Mathematically, let the unknown surface structure to be resolved along the *z*-axis (beam-propagating direction) shown as the green areas in Fig. 1 ▸(*b*) be denoted as **s** = [*s*_1_,…, *s*_*N*_]^T^, and the aperture structure at scanning position *p*_*m*_ be represented by 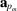 = 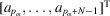, where the coefficients indicate the transmissivity at each location of the coded aperture. Here, *N* represents the number of discrete segments in the piecewise-constant representation of the entire aperture, while *m* and *M* are the indices and the number of scanning positions.

The scattering signal on the detector depends on both the aperture structure and the sample structure. As the aperture scans, different areas of the sample are probed, thus resulting in different detected signals. We can thus formulate the following system of equations,
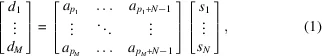
where **d** = [*d*_1_,…, *d*_*M*_]^T^ represents the intensity vector obtained from a single detector pixel after translating the coded mask *M* times. The positions of the coded aperture are tracked by the vector **p** = [*p*_1_,…, *p*_*M*_]^T^, which are determined accurately by the encoders of the linear motor during the aperture scanning process. This system can be compactly expressed in matrix form as
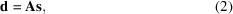
where **A** is the coding matrix with dimensions *M* × *N*.

In fundamental terms, the image reconstruction problem can be formulated as a least-squares optimization. Given the measured intensity data vector **d** and the system matrix **A**, which represents the linear relationship between the unknown signal vector **s** and the measured data, the problem is expressed as 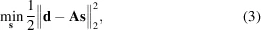
where 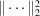 denotes the Euclidean norm. The matrix **A** encapsulates the relationship between the coded aperture pattern and the scattered X-ray signals into a pixel.

For a given detector pixel or detector region, the scanning-aperture measurements enable equation (3) to be solved, thereby reconstructing the sample structure associated with that detector pixel. In other words, for a pixel or a Bragg peak on the detector, the scanning-aperture data (**d** obtained by applying **A**) can be used to reconstruct the spatial distribution of the sample regions (**s**) contributing to that scattering peak. Moreover, by applying equation (3) to each detector pixel, the corresponding spatial distribution within the sample can be reconstructed, thereby establishing a mapping between detector pixels and sample pixels. Consequently, the results can also be interpreted in the reciprocal manner: for each sample pixel, the corresponding 2D scattering pattern can be determined. The resolution of the reconstructed image is not determined by the discretization parameter *N* but instead by the rank of **A**, the inverse problem formulation of equation (3), and the reconstruction solver. The precise aperture geometry was characterized using scanning electron microscopy (SEM), which is important for accurately constructing the matrix **A** and consequently influences the fidelity of the reconstructed **s** as well as the resulting imaging resolution. In summary, SI-GID combines imaging and scattering, enabling retrieval of 2D scattering data for each sample pixel. This allows the visualization of local sample regions at sample-pixel resolution, including features such as substrate areas or domains with distinct material orientation.

### 2.2. Experiment setup

We validated the SI-GID technique through 2D structural imaging of organic semiconductor thin films, a model system that highlights the importance of understanding the structure–property–performance relationship in functional materials. In organic electronics, particularly in the design of transistors, correlating thin-film structures with device performance is critical for optimization (Giri *et al.*, 2014 ▸; Lee *et al.*, 2012 ▸). Localized structural details of thin films such as domain shape, orientation, and polymorphism, which are important for advancing material design, can be difficult to capture due to the long footprint in grazing-incidence methods or limitations imposed by the beam size.

The organic films were prepared by casting a 1:1 mixture of C8- and C12-BTBT ([1]benzothieno[3,2-b][1]benzothiophene) in the melt state and cooling at 0.1° min^−1^, and formed millimetre-sized domains on silicon substrates pre-coated with a 100 nm polymethyl methacrylate layer. These films exhibited a uniform out-of-plane orientation across domains, while in-plane orientation varied between domains. The variations in in-plane orientation and polymorphism near localized domain boundaries is particularly critical for understanding and optimizing device performance but existing methods cannot provide such information.

**Figure 2:**
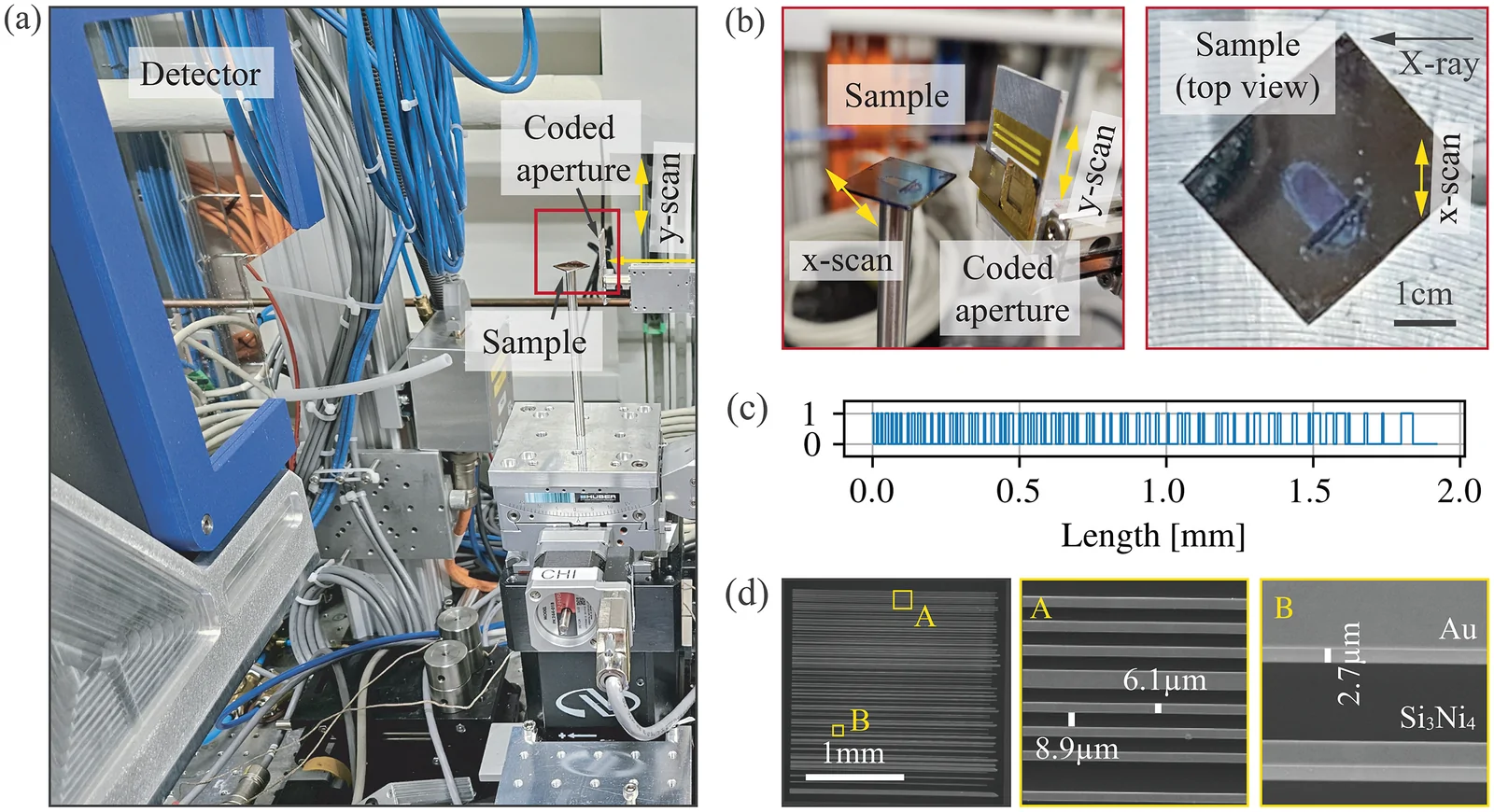
(*a*) Schematic of the setup showing the coded aperture, sample, and detector, with a zoomed view of the sample and aperture. The coded aperture is scanned vertically along the *y*-axis. (*b*) Optical photographs of the sample. The sample is scanned along the *x*-axis. (*c*) 256-bit coded aperture design, with the varying aperture direction placed along the *y*-axis. (*d*) SEM images of the fabricated aperture with zoomed-in views of regions A and B.

Experiments were conducted at the Complex Materials Scattering (CMS) beamline (11-BM) at the National Synchrotron Light Source II (NSLS-II), leveraging the standard GIWAXS setup with an additional coded aperture for structured illumination. The general layout of the setup is illustrated in Fig. 2 ▸. A 15 keV X-ray beam with size 200 µm (H) by 100 µm (V) illuminated the sample at a 0.3° incident angle, leading to a surface illumination of 200 µm (H) by 19 mm (V) on the sample. A coded aperture with a de Bruijn sequence of 256 bits, fabricated via direct-write lithography, introduced measurement diversity. Positioned 30 mm upstream of the sample, the aperture was scanned vertically over 600 µm in 1 µm steps, achieving a *z*-axis pixel size of 1 µm/sin (0.3°) = 190 µm on the sample.

**Figure 3:**
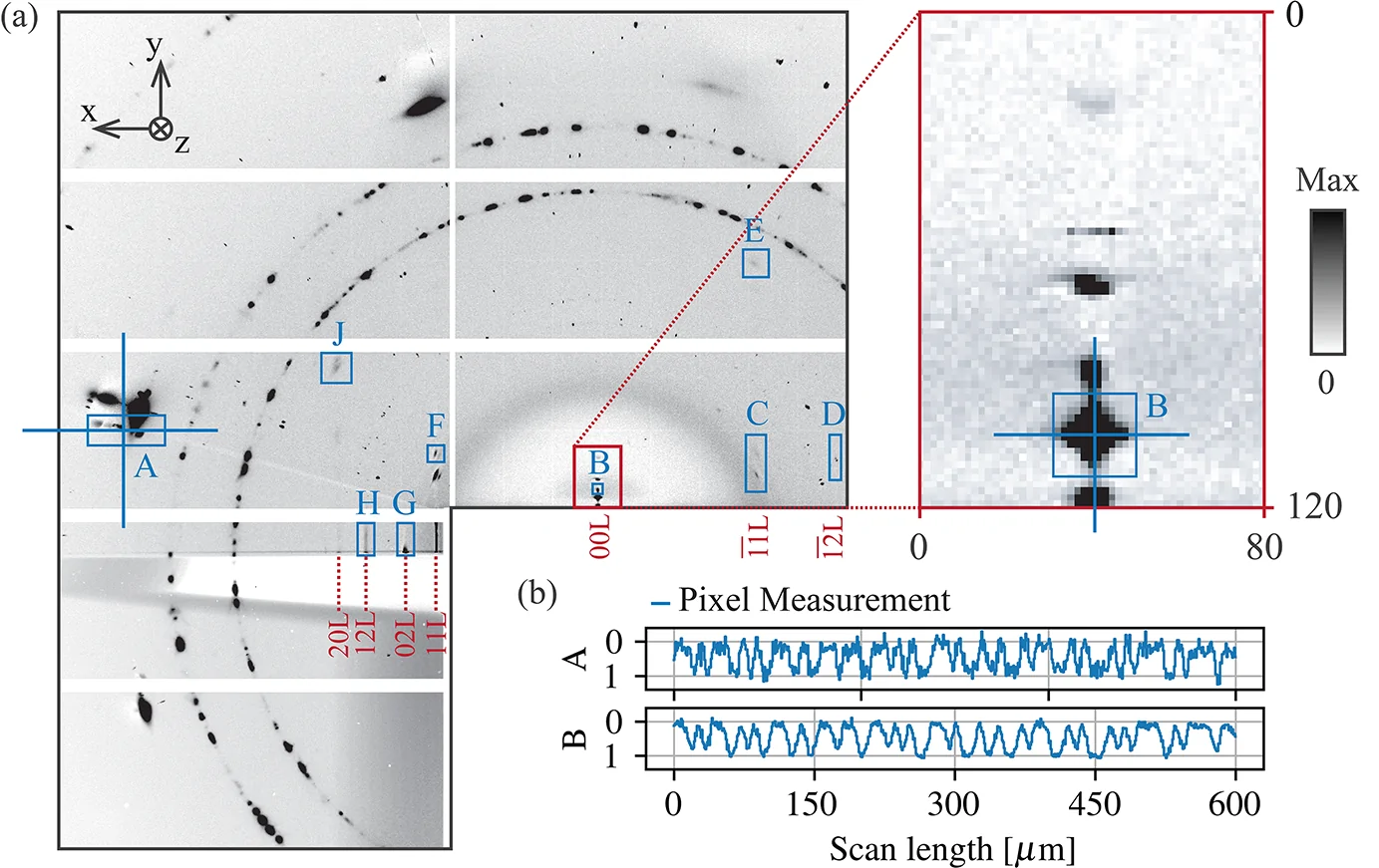
(*a*) Representative image (average of collected frames) recorded using a Pilatus800k detector. The analyzed peaks are labeled in blue, ranging from A to J. For every detector pixel, irrespective of scattering intensity, the corresponding spatial distribution within the sample can be reconstructed. Depending on the application, either the integrated ROI intensity or a single detector pixel can be used; in this work, detector pixels were used. (*b*) Measured intensities corresponding to detector pixels A and B obtained by scanning the coded aperture over a range of 600 µm.

**Figure 4:**
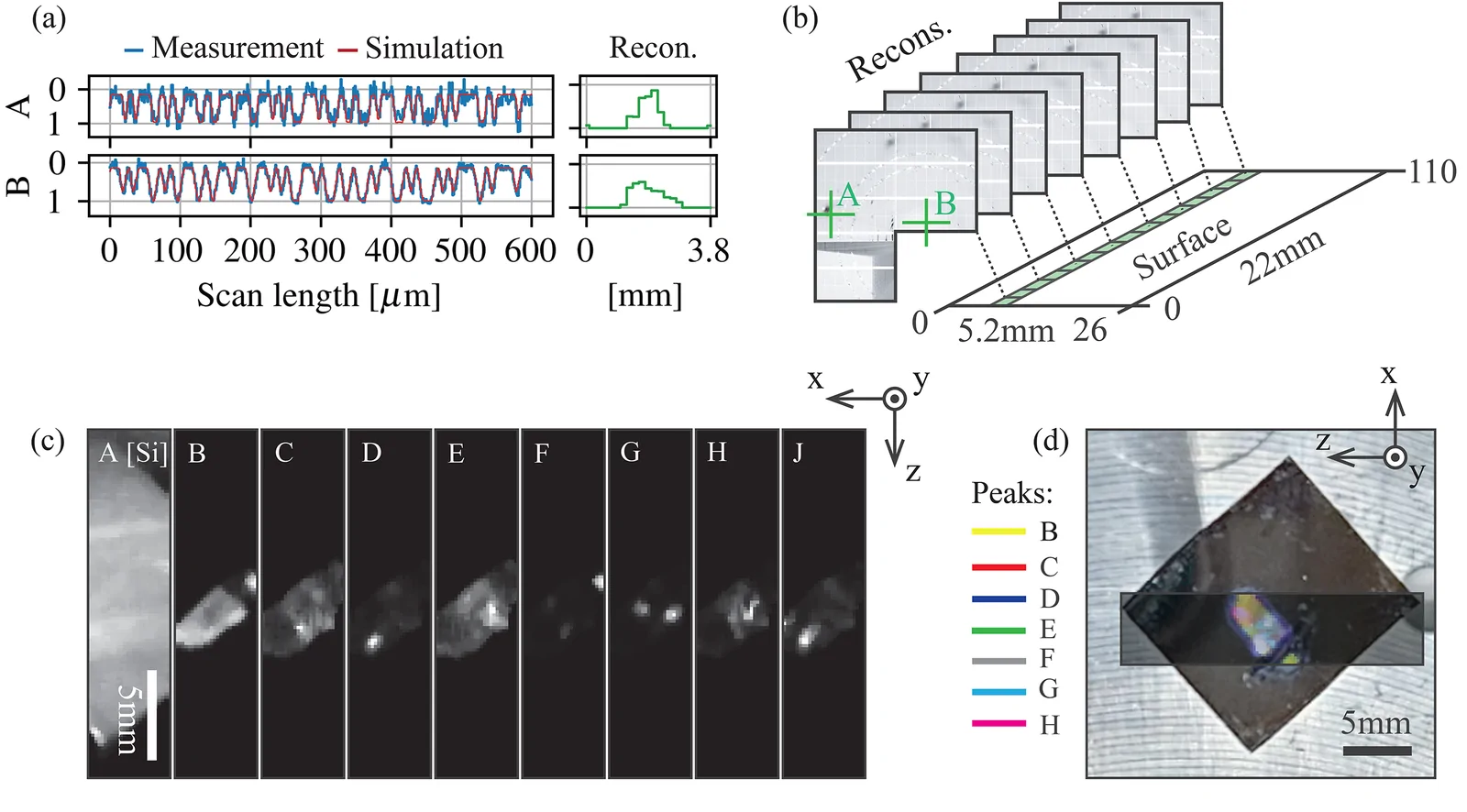
(*a*) Reconstructed signals for detector pixels A and B, compared with simulations and experimental data, showing good agreement. (*b*) With SI-GID, the scattering pattern at each sample-pixel can be determined, providing *N* scattering patterns over the area instead of one scattering pattern as in traditional GI methods. This capability is important because it enables spatially resolved scattering characterization that is not fundamentally limited by the beam size, allowing local structural information to be reconstructed from within a broadly illuminated region. (*c*) Reconstructed surface images for peaks identified in Fig. 3 ▸. Despite the weak scattering signal for some peaks, analysis can still be performed. (*d*) Overlay of selected reconstructed peaks on the optical image, highlighting spatial alignment.

The scattered X-rays were captured using a Pilatus800k detector with a pixel size of 172 µm, positioned 0.17 m downstream of the sample. A representative intensity recording obtained from this setup is shown in Fig. 3 ▸. The blue boxes are included for illustrative purposes, while the analysis in this work is based on the central pixel in A–J for demonstration. Depending on the application, an integrated intensity over a region of interest (ROI) may be used instead. Detector pixel A detects signals from the silicon substrate, while pixel B corresponds to signals scattered from the thin film sample. The corresponding measurement data for both pixels, as a function of the aperture scan, are presented in Fig. 3 ▸(*b*). With the measurement data at a detector pixel (**d**) and given aperture (**A**), the sample structure (**s**) can be reconstructed via equation (3). In other words, the experimental intensity modulations induced by the coded aperture scanning should align well with the simulated intensity variation introduced by the interaction between the coded aperture and the reconstructed sample, as shown in Fig. 4 ▸(*a*).

To obtain 2D sample information, horizontal *x*-scans of the sample were performed in 200 µm steps, matching the beam spot size, over a 5.2 mm range, resulting in 26 data points. Data were collected with an exposure time of 1 s per scan, synchronized with the stepping motion of the coded aperture. Digital reconstruction utilized non-negative least-squares optimization (Lawson & Hanson, 1995 ▸), implemented in *SciPy* (Virtanen *et al.*, 2020 ▸), to resolve scattering data across the beam footprint, enabling high-resolution spatial mapping of the sample’s surface structure.

## 3. Results

The reconstructed signal, **s** in equation (1), from a single detector pixel readout is shown in Fig. 4 ▸(*a*) with the corresponding simulated signal, demonstrating good agreement with the measurement [**d** in equation (1)]. By repeating the reconstruction process for each detector pixel, 2D scattering patterns can be reconstructed for each sample-pixel, as shown in Fig. 4 ▸(*b*). In other words, this enables a direct correspondence between real-space and reciprocal-space information: for any given scattering feature, such as a diffraction peak, the spatial distribution of the feature across the sample can be determined. This also means that we can visualize the sample regions (sample pixels) that contribute to the scattering feature. In contrast to conventional grazing-incidence methods, which yield only one averaged scattering pattern, this approach provides a full two-dimensional scattering pattern for each sample-pixel. This capability enables the investigation of thin-film inhomogeneities and grain boundaries with spatial resolution. Access to local thin-film structural information can provide valuable insights into structure–property relationships and offers design principles for optimizing functional material performance.

SI-GID with translational scanning can further provide visualization of local features across the entire thin film. Detector pixel A corresponds to the diffraction peak of the Si substrate, and the reconstructed signal reveals the spatial distribution of Si along the X-ray footprint in the *z* direction. With translational scanning in *x*, the combination of reconstructions at multiple *x*-positions gives the Si distribution map shown in the first panel of Fig. 4 ▸(*c*). The matching features from the silicon substrate between the optical image in Fig. 4 ▸(*d*) and the reconstructed image provide validation of our method.

This approach can effectively resolve both in-plane and out-of-plane structural variations associated with the targeted peaks. We selected several high-intensity scattering peaks identified in Fig. 3 ▸ and depicted their corresponding reconstructions in Fig. 4 ▸(*c*). The thin film sample displays a uniform out-of-plane orientation but varying in-plane orientations across different domains. Detector pixel B corresponds to the out-of-plane peak with the corresponding reconstruction illustrated in Fig. 4 ▸(*c*), which reflects the overall shape of the thin film. This demonstrates strong alignment with the optical photograph of the sample presented in Fig. 4 ▸(*d*). Detector pixels C to J correspond to distinct peaks associated with in-plane orientations, resulting in the visualization of different domains in Fig. 4 ▸(*c*). Even for weak scattering peaks or low-intensity detector pixels, the method remains applicable for reconstructing the corresponding spatial distribution on the sample. The exact in-plane orientations can be determined from the lattice and GIWAXS indexing, as shown by Tsai *et al.* (2021 ▸).

## 4. Discussions

We have presented SI-GID as a structured illumination method for GIWAXS that enables surface and near-surface imaging of planar samples without requiring sample rotation. Conventional GI X-ray scattering methods typically provide structural or morphological information averaged over large sample areas due to the elongated X-ray beam footprint. While tomographic methods combined with GIWAXS can provide localized information (Tsai *et al.*, 2021 ▸), it demands precise rotational alignment and complete tomographic datasets. Insufficient angular sampling or missing angles in tomography introduce tomographic reconstruction artifacts and compromise resolution.

SI-GID overcomes the limitations of existing methods by employing a scanning coded aperture to generate structured illumination, allowing direct reconstruction of localized structural information. For each sample-pixel, scattering patterns containing structural or morphological details are reconstructed, enabling the study of inhomogeneity and polymorphism. This sample-pixel-level information on crystalline structure, composition, and orientation may elucidate structure–performance relationships. This work shows potential for future development towards micrometre-scale domain resolution through, for example, optimization of the aperture structure, scanning strategy, and, ultimately, the inverse-problem formulation and reconstruction procedure. Materials previously studied with standard grazing-incidence methods, such as perovskites for solar panels, can benefit significantly such capability. For instance, resolving micrometre-sized domain orientations provides valuable insights for designing efficient solar cells (Li *et al.*, 2022 ▸; Sidhik *et al.*, 2024 ▸).

Compared with knife-edge or micro-beam scanning (Li *et al.*, 2012 ▸; Roth *et al.*, 2003 ▸), SI-GID encodes spatial information via structured illumination combined with computational reconstruction, thereby transferring part of the experimental complexity from beam focusing and high-precision mechanical control to the inverse reconstruction problem. In practice, focused micro-beams require specialized optics that may not be readily available at beamlines and can be challenging to implement. By contrast, SI-GID can be implemented without beam focusing or modifications to the sample stage, requiring minimal changes to existing beamline setups and configurations. For beamlines equipped with micro-beam capabilities, stable scanning stages, and strong sample scattering signals, the advantage of our method may therefore be less pronounced for the resolution demonstrated here. It is, however, possible to optimize the aperture pattern, scanning strategy, and computational methods to further improve the resolution. Overall, SI-GID provides a complementary approach to micro-beam diffraction techniques rather than a replacement.

The advantage of aperture scanning compared with full sample rotation in tomography is that it does not interfere with the operation or design of the *in situ* setup. By removing the need for a rotation stage, SI-GID is compatible with existing sample environments and custom experimental setups, while also improving measurement stability by avoiding rotational drift and vibration. In principle, it can therefore support *in situ* experiments, where full sample rotation is difficult or impossible to implement. However, temporal resolution remains the key limitation for probing fast *in situ* dynamics and thus constrains the types of experiments that can be performed. In the demonstration shown here, the 600 µm aperture scan with 1 µm step and 1 s exposure led to around 10 min per *x*-position. A possible experiment is to probe electrochemical processes with minute time resolution to study interfacial phenomena such as structural evolution or thin-film ordering. In the future, the number of scanning steps may be optimized to reduce acquisition time to enable broader applications. Moreover, while SI-GID may miss crystalline domains that do not satisfy the Bragg condition, this limitation can be mitigated by combining the method with a small number of rotational scans for comprehensive domain orientation analysis. Depending on the material lattice and sample–detector configuration, in particular the accessible *q*-range, either a small-range rocking scan or a few-angle tomographic approach can be performed over a narrow angular range to probe multiple domains. In contrast, tomography methods typically require large rotation ranges, often up to 180°, whereas the hybrid SI-GID approach may enable efficient orientation analysis with minimal rotation.

## 5. Conclusion

With its experimental stability, compatibility with existing sample environments, and ability to deliver spatially resolved scattering information, SI-GID provides a robust and versatile alternative to conventional grazing-incidence methods. By eliminating the need for sample rotation, SI-GID enables time-efficient, *in situ* studies of thin films, allowing dynamic processes to be probed on experimentally practical timescales that are substantially shorter than those required for full tomographic approaches. Furthermore, its spatially resolved diffraction capability provides strong potential for mapping localized variations in crystalline orientation and phase composition, offering insight into material inhomogeneity, polymorphism, and structure–property relationships in complex thin-film systems.

## Data Availability

Data underlying the results presented in this paper are not publicly available at this time but may be obtained from the authors upon reasonable request.
